# Embedding of Poorly Water-Soluble Drugs in Orodispersible Films—Comparison of Five Formulation Strategies

**DOI:** 10.3390/pharmaceutics15010017

**Published:** 2022-12-21

**Authors:** Denise Steiner, Marius Tidau, Jan Henrik Finke

**Affiliations:** 1 Institut für Pharmazeutische Technologie und Biopharmazie, Technische Universität Braunschweig, Mendelssohnstraße 1, 38106 Braunschweig, Germany; 2 Pharmazeutisches Institut, Pharmazeutische Technologie, Universität Tübingen, Auf der Morgenstelle 8, 72076 Tübingen, Germany; 3 Zentrum für Pharmaverfahrenstechnik (PVZ), Technische Universität Braunschweig, Franz-Liszt-Straße 35a, 38106 Braunschweig, Germany; 4 Institut für Partikeltechnik, Technische Universität Braunschweig, Volkmaroder Straße 5, 38104 Braunschweig, Germany

**Keywords:** orodispersible films, poorly water-soluble drugs, nanoparticle technology, lipid nanoparticles, amorphous solid dispersions, active pharmaceutical ingredients

## Abstract

The poor bioavailability of many newly developed active pharmaceutical ingredients (APIs) poses a major challenge in formulation development. To overcome this issue, strategies such as the preparation of amorphous solid dispersions (ASDs), and the application of the APIs in lipid nanocarriers or the wet-milling of the substances into nanoparticles have been introduced. In addition to an efficient formulation strategy, a dosage form that is accepted by all patients is also of great importance. To enable a simple application of the oral dosage form for all patients, orodispersible films (ODFs) are a very promising delivery platform for the APIs because the films directly disintegrate in the mouth. In this study, two poorly water-soluble APIs, fenofibrate and naproxen, were formulated using five different formulation strategies and then embedded in ODFs. It was found that the deliverable amount of API with one ODF highly depends on the formulation strategy as well as the physicochemical properties of the formulated API. The most promising film formulations were ASD-ODFs as well as films with API-loaded lipid nanoemulsions. Both showed a reduction of the dissolution time of the APIs from the ODF compared to an ODF with unformulated API micro particles. In addition, short disintegration times were achieved, although the mechanical film properties were slightly worse compared to the API-free film formulation.

## 1. Introduction

The increasing number of innovative dosage forms shows that the needs and demands of the patients have become more important in recent years, because a compliant intake of the medication and thus high patient acceptance is essential for a successful therapy. Several studies examined the most common treatment process attributes, such as route of administration [1]. As clear trend, oral administration was reported to be the most preferred, although forms such as tablets and capsules have to be swallowed as a whole to ensure full therapeutic effects [2]. However, it is known that this can cause problems, especially for children and elderly people [3,4]. To overcome these difficulties, formulation development is increasingly focusing on new and more advanced dosage forms such as orodispersible films (ODFs). ODFs are designed to dissolve directly in the oral cavity, allowing the embedded active pharmaceutical ingredient (API) to be swallowed with saliva [5,6]. In addition, films are very advantageous in terms of individualized solid dosage forms, e.g., by adjusting the size of the films to the required API dose according to the patient’s needs [7]. Due to the small volume of the films, the formulation of an API into the ODFs is quite challenging, regarding the limit in the dose, and in addition, special formulation strategies are required if the API is poorly water-soluble. Even when APIs are classified as poorly water-soluble, they may have different material attitudes which require a tailored formulation for each substance embedded in the ODF to enable an optimized API load.

One approach to formulate the poorly water-soluble APIs in ODFs is their embedding in a particulate form. Therefore, both micro as well as nanoparticles were closer investigated. While the embedding of microparticles resulted in rough film surfaces, especially at high API contents [8], the studies indicated that the API was released much faster from ODFs containing nanoparticles [9,10] due to their higher specific surface area and thus higher dissolution rate [11]. With these films, high loadings of up to 50 wt.% nanoparticles could be realized while maintaining good mechanical film properties as well as reasonable disintegration times [12,13]. 

Another possibility to embed the poorly water-soluble APIs in the film matrix is to formulate ASDs. Additives such as organic acids [14] or co-solvents [15] can be used to improve the solubility of the APIs in the film casting mass. Tetrabenazine was formulated in ODFs in this way. Its solubility was improved by directly dissolving the API in a mixture of water and citric acid. Storage studies showed that HPMC (hydroxypropyl methyl cellulose) was a promising film-forming material to maintain the API in the amorphous state for at least 6 months [16]. In another study, ethanol and PEG (polyethylene glycol) were used as co-solvents to improve the solubility of the poorly water-soluble API phenytoin. Promising ODFs in terms of film properties such as disintegration time and mechanical properties could be prepared by using PVA (polyvinyl alcohol) as a film-forming polymer, but X-ray diffraction patterns of the films still indicated a crystalline nature of the API [17].

Recent studies have investigated the approach of formulating poorly water-soluble APIs in lipid dispersions and embedding those in the film matrix. This could be more beneficial, because it is assumed that a formulation in lipids could positively affect the bioavailability and adsorption of the poorly water-soluble drugs in the gastro intestinal tract [18,19] and reduce food effects [20]. So far, only melatonin particles have been dispersed in tristearin microparticles or hydrogenated castor oil and further processed into ODFs with maltodextrin used as the film-forming matrix [21]. In a feasibility study with API-free lipid nanoparticles, the loading capacity of the ODFs was investigated by gradually increasing the lipid content in the film matrix. There, the focus was to maintain the nanoparticulate properties of the lipid particles and to obtaining ODFs with good mechanical properties as well as acceptable disintegration times. Lipid contents of up to 54 wt.% could be realized when the lipid nanoparticles were embedded in an HPMC matrix [22].

**Figure 1:**
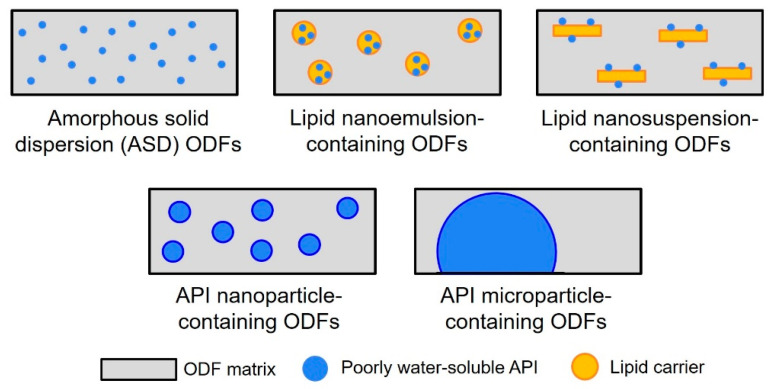
Formulation strategies for the embedding of poorly water-soluble APIs in ODFs.

The present study focused on the formulation of the following two poorly water-soluble APIs in ODFs: naproxen (NAP), as an already established model drug in formulation development and fenofibrate (FENO) because of its challenging lipophilic properties. Five different formulation strategies were closely investigated and are introduced in Figure 1: Preparation of amorphous solid dispersions (ASD-ODFs), embedding of the APIs in a lipid nanosuspension and a lipid nanoemulsion, embedding of API nanoparticles or micronized API particles (starting material) in the film-forming matrix. This work focused on evaluating the API content that could be loaded into one ODF, depending on the formulation strategy and compared the specific disintegration time, mechanical film properties and the dissolution behavior of the API-loaded ODFs of the different film types with each other.

## 2. Materials and Methods

### 2.1. Materials

Fenofibrate (FENO) and naproxen (NAP), both micronized, were used as poorly water-soluble APIs (Novartis Pharma AG, Basel, Switzerland, kind gift from the manufacturer). Some general characteristics of the APIs are given in Table 1. All films were prepared with the HPMC Pharmacoat 606 (hydroxypropyl methyl cellulose; Shin-Etsu Chemical Eo., Tokyo, Japan; kind gift from Harke Pharma, Mülheim an der Ruhr, Germany) and the plasticizer glycerol (Roth, Karlsruhe, Germany). The lipid carrier systems consisted of tristearin as the solid lipid (Dynasan^®^ 118; HülsAG/Cremer Oleo, Witten, Germany; kind gift from the manufacturer) and medium chain triglycerides as liquid lipid (MCT; Miglyol^®^ 812, Caesar&Loretz, Hilden, Germany). For the stabilization of the various nanodispersions, the following additives were used: vinylpyrrolidone-vinyl acetate copolymers Kollidon^®^VA 64 (KVA; BASF, Ludwigshafen, Germany), HPMC (Pharmacoat 606), sodium dodecyl sulphate (SDS; Roth, Karlsruhe, Germany). ODFs were prepared with distilled water and/or ethanol (purity 99.8 vol.%, Sigma-Aldrich, Taufkirchen, Germany). Sodium phosphate and hydrochloride acid (both Roth, Germany) as well as tetrahydrofuran (THF; Sigma-Aldrich, Taufkirchen, Germany) were used for characterization purposes. 

### 2.2. Preparation of Orodispersible Films

Due to the various formulation strategies, the ODFs were prepared in different ways. To allow a comparison of the films, the target dry film weight for all ODF types was 10 mg cm^−2^. After film preparation, all ODFs were packed into polymer bags and stored at 20 °C (40 % relative humidity) prior to characterization. 

#### 2.2.1. Amorphous Solid Dispersion (ASD)-ODFs

ASD-ODFs were prepared with four different API-concentrations. Therefore, HPMC (15 wt.%) was first dissolved in a water/ethanol mixture (ratio 1:2) with a stirring paddle at 1320 rpm for 20 min. Afterwards, glycerin (5 wt.%, referred to the total solution mass) was added and the solution was further stirred for 10 min. FENO or NAP were then added to the solution to obtain API-loads in the dried ODFs of 2 wt.%, 4 wt.%, 8 wt.% and 16 wt.%. The APIs were directly dissolved in the HPMC-solution under stirring for an additional 15 min. The film casting mass was cast with an automatic film applicator coater (ZAA 2300; Zehntner, Sissach, Switzerland) onto a PET (polyethylene terephthalate) foil using a height-adjustable doctor blade (width 200 mm) with a gap height h_gap_ = 800 µm and a casting speed of 10 mm s^−1^. The ASD-ODFs were dried at 30 °C for 3 h in an oven.

#### 2.2.2. ODFs Containing API-Loaded Lipid Nanodispersions

Two different kinds of lipid nanocarriers were loaded with APIs in this study: solid lipid tristearin nanosuspensions and liquid lipid MCT nanoemulsions. Both carrier systems were first prepared without API by high-pressure homogenization (Microfluidizer M110-P; Microfluidics, Westwood, MA, USA). Depending on the lipid type, the process temperature was room temperature (MCT nanoemulsion) or approximately 85 °C (tristearin nanosuspension). The nanodispersions of both lipids were stabilized with the surfactant SDS. Thus, an aqueous solution containing SDS (0.25 wt.%) was first prepared and tempered to the process temperature. Afterwards this solution was added to the liquid (for tristearin, molten) lipid and a pre-emulsion was prepared by homogenizing the two phases with a T25 digital ULTRA TURRAX^®^ (IKA, Staufen im Breisgau, Germany) for 2 min at 13,000 rpm. The high-pressure homogenization was then performed at the corresponding process temperature at 800 bar in 10 cycles. Afterwards, both formulations were cooled down to room temperature, which caused the nanosized tristearin droplets to crystallize in the dispersion. 

The lipid carrier nanodispersions were loaded with FENO or NAP using the passive loading method [24]. To enable a maximum API load of the tristearin formulation, 25 g of the nanosuspension were filled in a glass vial and an excess amount of API (approximately 0.2 g) was added. The mixture was stirred at 50 rpm at 20 °C for 5 days. The formulation was then filtered through a 0.45 µm PVDF filter (Roth, Karlsruhe, Germany) to remove excess API crystals from the tristearin suspension. The MCT nanoemulsion was loaded with FENO and NAP in the same way. While an excess amount of API (approximately 0.3 g) was added to 15 g of the nanoemulsion to achieve maximum API loading, an exact amount of 0.06 g FENO powder was added to 15 g of the nanoemulsion to prepare a formulation with a FENO-amount of 4 wt.% referred to the lipid content in the nanoemulsion. After an incubation time of 5 days (at 20 °C), the emulsions were also filtered through a 0.45 µm PVDF filter. All nanodispersions loaded with FENO or NAP were stored at 20 °C for one day before being further processed into ODFs. 

For film preparation, the API-loaded lipid dispersions were mixed with an aqueous film-forming solution. This was prepared by adding HPMC (10 wt.%) and glycerol (3.3 wt.%) to approximately 80 °C hot water. The HPMC solution was then allowed to cool down to room temperature while stirring for at least 8 h until all air bubbles had disappeared. Preliminary studies determined the maximum lipid content that can be embedded in the film matrix. There, the focus was on maintaining the nanoparticulate properties of the nanosuspensions under investigation after redispersing the films in water and achieving good mechanical properties and reasonable disintegration times. On this basis, the following lipid contents were embedded in the HPMC matrix: 49.6 wt.% tristearin nanoparticles (according to [22]) and 24.1 wt.% MCT droplets (Figure S1, Supplementary data). To enable a homogenous distribution of the lipid in the film matrix, the lipid dispersions were homogenized with the film-forming solution using an ULTRA TURRAX^®^ for 1 min at 10,000 rpm. The film casting mass was then poured into glass Petri dishes (diameter 10 cm). The formulations with the MCT nanoemulsion were oven-dried at 40 °C for 2 h and the tristearin containing films were dried overnight at room temperature. 

#### 2.2.3. Preparation of API Particle-Containing ODFs

ODFs containing API micro as well as nanoparticles were manufactured. Nanosuspensions were prepared by grinding the API particles in the stirred media mill MiniCer (kind loan of Netzsch Feinmahltechnik GmbH, Selb, Germany). The mill is equipped with a ceramic stirrer with pins and a sieve for grinding media separation and was operated in circuit mode with a stirrer speed of 9 m s^−1^. Yttrium stabilized zirconium oxide grinding beads with sizes between 300 µm and 400 µm (ρ_GM_ = 6067 kg m^−3^; Sigmund Lindner, Warmensteinach, Germany) and a filling ration of φ_GM_ = 0.8 were used. The process chamber and the storage vessel were water-cooled (T_water_ = 10 °C) during the process time. For both APIs, the solid content in the suspension was 5.2 wt.%. The particle stabilization against agglomeration was realized with a combination of a polymer and a surfactant for both APIs. NAP was formulated with KVA (25 wt.%) and SDS (2.5 wt.%) [12] and FENO with HPMC (34 wt.%) and SDS (3.4 wt.%, all refer to the solids content of the suspension). After a grinding time of 4 h, the nanosuspensions were removed from the mill and HPMC (15 wt.%) as well as glycerol (5 wt.%) were directly added to the nanosuspension and solved while stirring with a paddle stirrer for 30 min. The targeted particle concentration in all films was 4 wt.% in order to enable a comparison to the other ODFs. However, previous studies already indicated that up to 50 wt.% of NAP nanoparticles in the ODFs are feasible [12]. After degassing the film casting mass overnight, it was cast with the automatic film applicator coater with a gap height h_gap_ = 750 µm, and the films were dried in an oven at 50 °C for 2 h. 

Microparticle-containing ODFs were prepared by directly adding the API particles (starting material) into the degassed film-forming solution (10 wt.% HPMC and 3.3 wt.% glycerol). The targeted particle concentration in the dry films was also 4 wt.%. For a good particle distribution, the microparticles were homogenized with an ULTRA TURRAX^®^ for 0.5 min in the film-forming solution, poured into a glass Petri dish (diameter 10 cm) and dried at room temperature overnight. 

#### 2.2.4. HPMC Unladed-ODFs

Unloaded-ODFs were manufactured for comparison purposes. They were prepared from a film solution containing 15 wt.% HPMC and 5 wt.% glycerol, both dissolved in approximately 80 °C hot water and cooled down to room temperature while stirring. The film-casting mass was degassed overnight and cast into ODFs with the automatic film applicator coater with a gap height h_gap_ = 750 µm. The ODFs were dried for 2 h at 50 °C in an oven. 

### 2.3. Characterization Methods

#### 2.3.1. Particle Size Analysis

All nanodispersions as well as the API microparticles and lipid dispersions redispersed from the ODFs were measured by laser diffraction (LD). The ODF samples were prepared for the particle size measurements by redispersing a film with 4 cm^2^ in 0.9 mL saturated API-solution (for ODFs loaded with API micro or nanoparticles) or distilled water (for lipid-containing ODFs) for 1 h. All lipid-containing formulations were measured with the LD-device LA-960 (Horiba Scientific, Kyoto, Japan) in a fraction cell, and the API particle-containing formulations were measured with a Mastersizer 3000 (Malvern Panalytical, Malvern, UK) using a Hydro MV dispersing unit (stirrer speed 2000 rpm). Each sample was measured in triplicate. All LD measurements were calculated according to the Mie theory-based evaluation model. Dependent on the composition of the samples, the refraction and absorption indices were adjusted. 

#### 2.3.2. Characterization of API Content

ASD-ODFs as well as films containing API micro and nanoparticles were directly dissolved in water to evaluate the API content by UV-Vis spectroscopy. Therefore, circular samples were punched out of the films with a diameter of 12 mm and solved in 100 mL phosphate buffer (pH 6.8) for 2 h for NAP-containing ODFs and overnight for FENO-films. The API content was then determined with a SPECORD 210 PLUS (Analytic Jena, Jena, Germany) at a wavelength of 331 nm for NAP-samples and 287 nm for FENO-samples. 

Lipid-containing ODF samples were first disintegrated (as 4 cm²) in 1 mL water for 1 h to dissolve the film forming polymer and disperse the lipid nanoparticles. Then, 100 µL of the dispersion of the ODF was diluted in 10 mL of a THF/water mixture (ratio 9:1, *v*/*v*) to dissolve the lipid particles. The FENO and NAP concentrations in the lipid-containing ODFs were then determined by UV-Vis spectroscopy using a SPECORD 40 (Analytic Jena, Jena, Germany). The measurements were performed at a wavelength of 287 nm for FENO-containing and 233 nm for NAP-containing samples. To evaluate the API content of the lipid-containing ODFs, the absorption of the unloaded lipid nanodispersion was subtracted as a blank value from the samples. 

Prior to all measurements, the solvent was used as baseline and a calibration curve was prepared for both APIs.

#### 2.3.3. Analysis of Crystallinity

The crystalline state of the starting materials and the ODFs was characterized by X-ray diffraction (XRD). ASD-ODFs were measured with an Empyrean CU LEF HR Goniometer equipped with a PANalytical PIXcel-3D detector (Netherlands) and the lipid-containing ODFs were measured with a PW3050/60 MPD Goniometer equipped with a Pre FIX X’Celerator detector (PANalytical, EA Almelo, Netherlands). All samples were measured with a step size of 0.05° 2θ in a range from 5° to 45° 2θ.

ASD-ODFs were additionally examined with a polarizing microscope to evaluate potential crystals formed during the manufacturing process. A Leica DMLM (Leica, Wetzlar, Germany) was used in combination with a DP12 camera system (Olympus, Hamburg, Germany). 

#### 2.3.4. Determination of Melting Events

Differential scanning calorimetry (DSC) was used to determine the melting events of the API starting materials as well as the APIs and lipids embedded in the ODFs. When powders were characterized, they were weighed directly in the aluminum crucibles (40 µL). For ODFs, samples with a diameter of 4 mm were punched out and three films were placed on top of each other in the aluminum crucibles. After filling, they were cold welded and analyzed with a DSC3+ equipped with a FRS6+ sensor (Mettler Toledo, Columbus, OH, USA). All samples were measured with a heating rate of 10 °C min^−1^ starting at 25 °C up to 170 °C. 

#### 2.3.5. Mechanical Film Properties

The mechanical film properties were measured with the material testing machine ZwickiLine+ Z2.5 equipped with pneumatic sample holders and the force transducer Xforce HP with a maximum force of 50 N (Zwick Roell, Ulm, Germany). Dumbbell specimens with a width of 2.05 mm in the film center and 6 mm on the sides were punched out of the films. Before the film was measured, the thickness of each sample was determined with a digital micrometer screw (accuracy 0.001 mm). The samples were stressed until rupture at a clapping length of 17.5 mm with a testing speed of 5 mm min^−1^. The tensile strength was calculated for each sample from the maximum force before rupture and the cross-section area of the film. Furthermore, the elongation before breakage was determined. For each film formulation, six samples were prepared and analyzed independently and the mean values as well as the standard deviations were calculated. 

#### 2.3.6. Determination of Specific Disintegration Time

The SFaB (slide frame and ball) method introduced by Steiner et al. [12] and further optimized with an insert afterwards [25] was used for the determination of the disintegration time of the ODFs. Films with a size of 30 mm × 40 mm were prepared, and the sample thickness was measured at four different positions. Then, the samples were fixed in the frame, and 0.9 mL water was spread over the film surface and a stainless-steel ball (size 10 mm, weight 4 g) was placed on top of the film while the measurement time was started. To fix the ball in the middle of the film, an additional insert was placed on top of this setup. The film was considered as disintegrated when the ball fell through the film to the bottom of the device and the time was stopped. Due to the slightly different film thicknesses caused by the various formulation strategies, the specific disintegration time was calculated for all films by setting the disintegration time in correlation with the film thickness. For each film formulation, four samples were prepared, measured and the mean values as well as the standard deviations were calculated. 

#### 2.3.7. Dissolution Behavior of API Embedded in ODFs

The dissolution behavior of all API-loaded ODFs was measured with an automated flow-through cell apparatus (CE 7smart; Sotax, Aesch, Switzerland). The design was chosen following Sievens-Figueroa et al. who evaluated different experimental setups for dissolution studies in a flow-through cell with ODFs containing nano and microparticles [26]. A closed loop was chosen for the measurements. A sample was pumped from the storage vessel through the automatic UV-VIS spectrophotometer (SPECORD 200 Plus; Analytic Jena, Jena, Germany) and through the cells (internal diameter 22.6 mm). The cells were equipped with a 5 mm ruby ball, functioning as a check valve with the bottom of the cone, to prevent material to descend into the inlet tubing. To enable a homogeneous flow through the cell and a securing of the film, 3 g glass beads with a diameter of 1 mm were placed in the cell first. Then, the ODF sample (diameter 12 mm) was placed on top of these. To hold the ODF in position, further glass beads (2.5 g) were added to cover the film. To ensure that aggregates of particles or lipids as well as clusters of disintegrated film do not leave the cells, two different filters were used. The first filter was a microfiber glass filter with a pore size 2.7 µm (GF/D, GE Healthcare, München, Germany) and the second a cellulose filter with a pore size 0.2 µm (Sartorius, Göttingen, Germany). NAP-containing ODFs were dissolved in 100 mL phosphate buffer (pH 6.8) and FENO-ODFs in 150 mL phosphate buffer (pH 6.8) containing 0.1 mol L^−1^ SDS. The dissolution behavior was investigated for 90 min for all ODFs. During the dissolution experiments, the temperature was maintained at 37 °C ± 1 °C and the flow rates were 8 mL min^−1^, while the stirring speed in the storage vessels was 200 rpm. The API concentration was automatically measured by the inline UV-Vis spectrophotometer each minute at a wavelength of 287 nm for FENO and 331 nm for NAP. 

## 3. Results and Discussion

In the following section, the ASD-ODFs, the lipid-containing films and the ODFs with embedded API micro and nanoparticles are first considered separately. Afterwards, the API-containing films formulated with the five different strategies were compared to each other in terms of their specific disintegration time, mechanical properties and dissolution behavior. 

### 3.1. Characterization of API-Loaded ASD-ODFs

**Figure 2:**
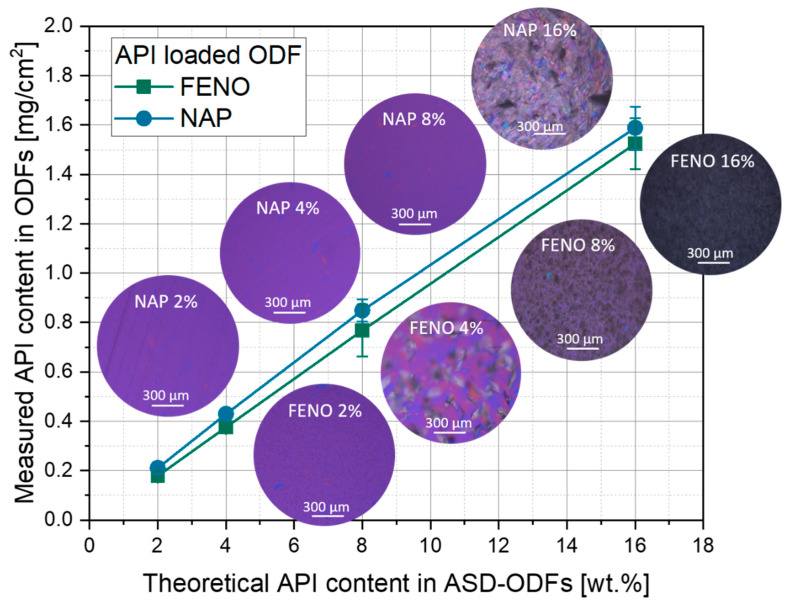
Comparison of measured and theoretical API content in ASD-ODFs and polarized light microscopic images of the corresponding films; n = 3.

When APIs are formulated as ASD-systems, their solubility in the matrix is important to prevent crystallization during film drying. To investigate the degree to which NAP and FENO can be incorporated into the HPMC film matrix, ASD-ODFs were prepared with four different API concentrations (2, 4, 8 and 16 wt.%, referred to the dry film weight). In the first step, the measured API content of these films was compared with their theoretical content and polarized light microscopic images of the films were taken (see Figure 2). Assuming that all films had a surface weight of 10 mg cm^−2^, the measured API content in ODFs with 2 and 4 wt.% API differs only slightly from the theoretical content, independent of the embedded API. With a further increase of the API content in the films, the deviation of the measured API content and the theoretical content increased, with clearly visible standard deviations for the NAP-containing ODFs with 16 wt.% and FENO-ODFs with 8 and 16 wt.%. When looking at the microscopic images, it can be observed that particulate structures can be seen in these films. It is therefore assumed that the drying of these films caused a recrystallization of the dissolved API and an inhomogeneous distribution of the embedded APIs, although no particles were observed in the film casting mass. The ODFs loaded with 4 wt.% FENO showed only few but larger crystalline structures. In this case, it was assumed that the saturation concentration was slightly exceeded, leading to the formation of single crystallization nuclei and thus slow crystal growth during the drying process. No crystals were visible on the images with a FENO loading of 2 wt.%. 

Compared to the ODFs loaded with FENO, the loading capacity appears to be higher when NAP was used as model API in these films. The polarized light microscopic images showed no crystals in the film matrix up to a NAP content of 8 wt.%, while crystals were observed in the ODFs at an API content of 16 wt.%. For a more precise determination of the maximum loading capacity of the ODFs with both APIs, XRD measurements were made. 

**Figure 3:**
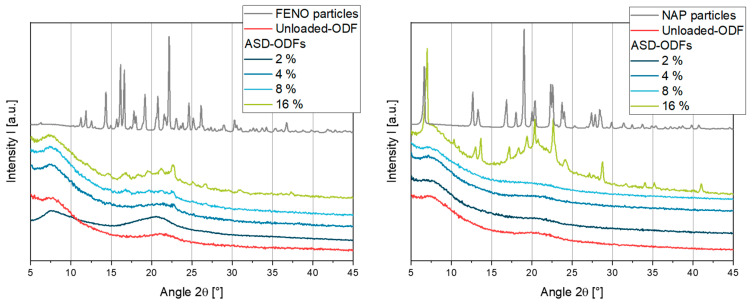
XRD measurements of ASD-ODFs loaded with the two model APIs FENO (**left**) and NAP (**right**).

The XRD diffractograms of the FENO and NAP starting materials indicated a crystallin state, while the unloaded-ODF composed of HPMC and glycerol is amorphous (see Figure 3). Several high intensity signals were seen in the diffractogram of the FENO particles, such as the reflex at 22.5° 2θ. This reflex could also be detected in the ASD-ODF with 16 wt.% FENO; in addition, smaller reflexes are also visible which indicate the presence of FENO particles. A comparison of this diffractogram with the amorphous unloaded-ODF show that the amorphous state of the film matrix could also be found in this film. As the FENO content in the films decreased, the intensity of the signals was reduced. While the diffractogram of the ODF with 2 wt.% FENO did not show any distinct FENO reflections, no clear conclusion could be drawn for the ODF with 4 wt.% FENO.

Similar to the diffractograms of the FENO-containing samples, the NAP starting material showed signals with strong intensities like the reflexes at approximately 7° and 19° 2θ. The crystalline state of NAP and the amorphous state of the film matrix were both found in the ODF with 16 wt.% NAP. However, when the NAP content decreased, no signals indicated a crystalline structure in the films. This shows that NAP could be embedded as an amorphous dispersion with API contents of up to 8 wt.% in these films.

**Figure 4:**
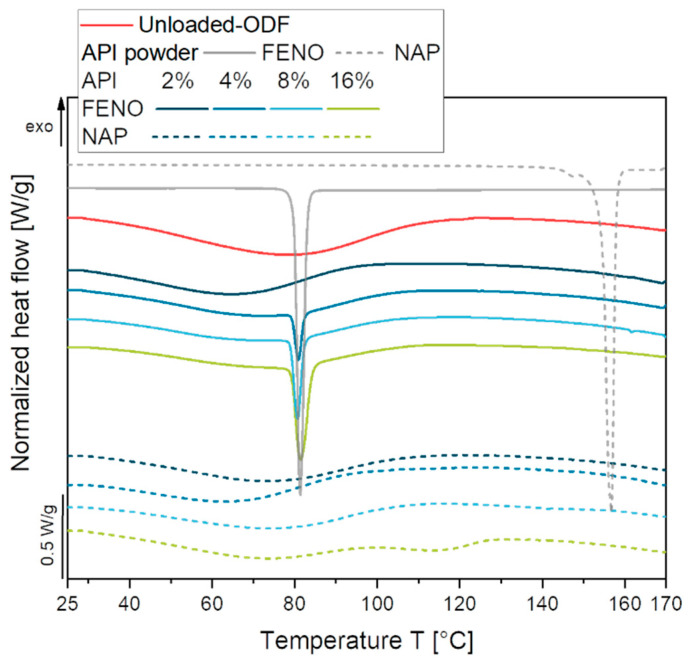
DSC melting curves of ASD-ODFs containing different concentrations of FENO and NAP, measured one day after film preparation.

In order to clarify the state of the API in the FENO-loaded ODFs, additional DSC measurements were performed on all films. The API starting materials show clear endothermic peaks at approximately 81 °C and approximately 158 °C indicating the melting of FENO and NAP, respectively (Figure 4). Closer examinations of the FENO-loaded ODFs revealed melting signals at approximately 81 °C for ODFs loaded with 4 wt.% FENO or more, indicating the presence of crystalline particles in these films. However, an amorphous state of the API was confirmed for the ODF loaded with 2 wt.% FENO.

Regarding the melting event for NAP-loaded APIs, the previous results of the XRD analysis were confirmed. NAP could be embedded in the film matrix up to an API content of 8 wt.% in an amorphous state. A broad endothermic peak was detected at approximately 115 °C for the ODF with 16 wt.% NAP. This event is assumed to be due to the melting or dissolution of the recrystallized NAP crystals within the softened HPMC film matrix, indicating a crystallization process during film preparation. 

Considering the knowledge gained in this part, two different poorly water-soluble model APIs were selected and embedded in the HPMC film matrix as ASDs. Both APIs are listed in the BCS (Biopharmaceutical Classification System) in class II but their ODF loading capacity differs significantly. For successful embedding of API molecules, intermolecular bonds with the matrix polymer are required to reduce the molecular mobility of the APIs and thus provide stability to the system [27]. For the APIs shown, it could be concluded that the intermolecular forces between the higher water-soluble NAP (loading capacity 8 wt.%) and the polymer matrix seem to be higher than for the lower water-soluble FENO molecules (loading capacity 2 wt.%, compare Table 1). For a deeper understanding of the differing molecular mobility of both APIs and thus intermolecular forces, additional methods like solid-state nuclear magnetic resonance spectroscopy will be considered for future studies. 

### 3.2. Characterization of Lipid-Containing ODFs

Another strategy to improve the bioavailability of poorly water-soluble APIs is their formulation into lipid nanodispersions and the embedding of the lipid carriers in a film-forming matrix. Another important aspect in this case is the maximum loading capacity of the lipids with the API under investigation. Therefore, the maximum achievable FENO and NAP load (target: max.) in the MCT nanoemulsions and tristearin nanosuspensions was evaluated. In addition, the MCT nanoemulsion was loaded with a targeted concentration of 4 wt.% FENO (see Table 2). 

After the incubation of the lipid dispersions with the APIs, the highest API concentrations in the lipids were obtained for the MCT nanoemulsions. The MCT droplets could be loaded with maximum 8.0 wt.% FENO and 3.2 wt.% NAP. The formulation with a targeted FENO load of 4 wt.% had a measured API loading of 3.8 wt.%. In comparison, a maximum API loading of 3.5 wt.% FENO and 2.7 wt.% NAP was achieved for the tristearin nanosuspensions. These findings confirmed the results of previous studies with FENO, that lower loading capacities were achieved for lipid nanoparticles compared to lipid emulsions [28,29]. The reason for this difference is the physical state of the lipid carrier. During the incubation of a nanoemulsion with the API particles, the API molecules can be loaded into the droplet matrix as well as its surface. When the lipid carrier consists of a crystalline lipid matrix, the probability of an API molecule to be loaded into the well-organized particle matrix is quite low. Therefore, the API molecules are mainly located only on the particle surface [30] as confirmed by fluorescence dye studies [31]. Even though tristearin particles crystallize in a platelet-like shape (when in β-modification) [32] and thus have a higher interfacial area than the spherical liquid lipid droplets, the loading capacity of the nanosuspensions is usually lower. 

Another aspect observed is the dependency of the loading capacity on the type of API. In this case, the physicochemical properties of the API play an important role. One feature to roughly estimate whether an API could be successfully loaded in the lipid matrix is the lipophilicity of the API, which is considered with the log P value. With a log P value of 5.2 for FENO, the loading capacity in the lipid carriers was higher than for NAP (log P 3.2). This displays the contrary trend to the case of ASD-ODFs where the interaction potential towards the water-soluble polymer HPMC was decisive and accordingly, the more hydrophilic NAP showed higher amorphous concentrations (Section 3.1).

When lipid carriers should be used as solid drug delivery systems, they must be embedded in a solid matrix while their nanoparticular properties should be maintained throughout the manufacturing process. In this study, the dispersions were embedded in a film-forming HPMC matrix. Based on previous studies, it was shown that a content of 49.6 wt.% tristearin nanoparticles (loaded with no API) could be embedded in the film matrix [22]. For the MCT nanoemulsion, a lipid content of 24.1 wt.% was chosen. Thus, the films loaded with the MCT nanoemulsion achieved an API concentration of 0.192 mg cm^−2^ (max. FENO) and 0.076 mg cm^−2^ (NAP) and when the MCT nanoemulsion was loaded with 4 wt.% API, 0.091 mg cm^−2^ of the FENO was in the film. Due to the high tristearin concentration in the ODFs, these films could be loaded with a total amount of 0.172 mg cm^−2^ FENO and 0.133 mg cm^−2^ NAP. 

**Figure 5:**
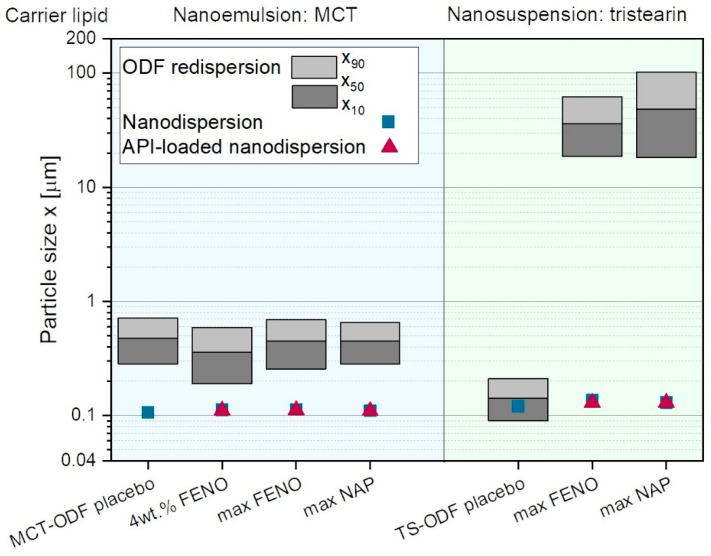
Particle sizes after redispersion of ODFs loaded with FENO and NAP containing MCT emulsion and tristearin suspension as well as API-free (square) and API-loaded (triangle) lipid dispersions (“MCT-ODF placebo” for nanoemulsion and “TS-ODF placebo” for nanosuspension).

To determine if the APIs loaded in the lipid carriers had an influence on the particle sizes after the redispersion of the films in water, the droplet and particles sizes of the lipids were measured (Figure 5). For comparison purposes, dispersions loaded with the APIs before embedding in the HPMC matrix were also measured, as well as placebo lipid-ODFs. The placebo films only embedded the nanoemulsion and suspension, respectively, but no API was loaded in the lipid carriers (named “MCT-ODF placebo” for nanoemulsion- and “TS-ODF placebo” for nanosuspension-containing ODFs). Particle size measurements of the nanodispersions loaded with API showed no significant changes in particle fineness after the loading process. 

The particle sizes after redispersion of the MCT-loaded ODFs increased for all films, including the placebo film, compared to the nanoemulsion. For the placebo formulation, a particle size increase of approximately 350 nm (x_50_-value) was observed. This increase was believed to be caused by the lipid droplets coalescing during the drying process. Similar particle sizes were obtained when the ODFs with API-loaded nanoemulsions were dispersed in water. In this case it could be concluded that the increase in the particle size is mainly caused by the physical state of the droplets and not by the embedded APIs. 

A different behavior was seen for the tristearin-loaded ODFs. While the redispersion of the TS-placebo ODF showed only a small increase in the particle sizes, large agglomerates were observed when the lipid particles were loaded with APIs. This indicated that the lipid particles could be sufficiently stabilized against agglomeration in the ODF matrix in absence of the APIs, as shown previously [22]. As soon as the nanosuspension was loaded with the APIs, the API molecules were mainly located at the surface of the lipid particles, as described above. This seemed to have a significant impact on the electrosteric stabilization mechanism of the lipid particles embedded in the solid matrix structure, since particle sizes larger than 10 µm occurred after redispersion of the films while no agglomeration was observed when the dispersions were loaded with APIs. The reason for the formation of agglomerates in the film-polymer matrix when the lipid particles were loaded with APIs needs to be investigated in future studies. 

**Figure 6:**
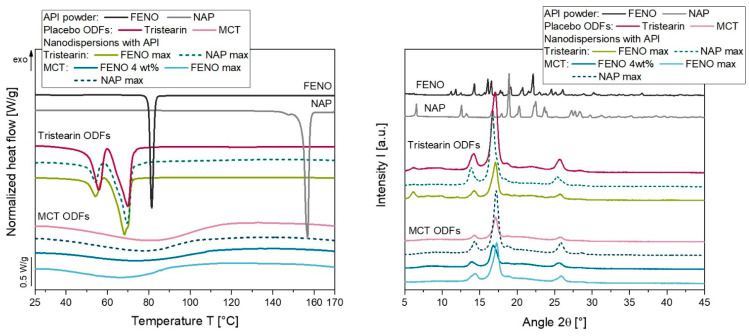
DSC melting curve (**left**) and XRD diffractograms (**right**) of API loaded lipid-containing ODFs. The DSC curves were normalized to the sample mass and the lipid content of the respective formulation for better visualization.

Recrystallization of the APIs loaded in the lipid nanodispersions is not desirable and must be prevented during the processing of the nanodispersions into ODFs. To identify crystalline API particles in the ODFs, DSC and XRD measurements were performed, which are shown in Figure 6. 

Films with embedded API-loaded MCT droplets did not show endothermic peaks that could be attributed to a melting event of the crystalline API particles, compared to the unloaded MCT placebo films. The situation was somewhat different when the heating curves of the tristearin-loaded ODFs were considered. Tristearin was embedded in the ODFs in a solid state and can crystallize in mainly two different modifications: the metastable α-form and the stable β-form [33,34]. The DSC measurements of the tristearin ODFs showed that both polymorphic forms were present in the ODFs, as indicated by the two endothermic peaks at approximately 54 °C (α-form) and 69 °C (β-form). Although no further melting events could be detected when the tristearin particles had been loaded with API, no conclusive statement about the state of the API in the films could be made in this case. During the constant heating of the ODFs and the associated melting of the tristearin particles, it could be possible that the API crystals present in the films would be dissolved in the liquid lipid during the measurement. Therefore, these API particles would not show up as an endothermic melting peak during the measurement. To ensure that no API crystals were present in the lipid-loaded ODFs, additional XRD measurements were performed.

A comparison of the diffractograms of the API-containing ODFs with the lipid-loaded but API-free placebo films showed that the location of the reflexes were similar in the corresponding films. Slight shifts of the peaks could be attributed to sample preparation when the films were fixed in the sample holder. None of the characteristic reflexes of the API particles could be detected in the film samples. It could be concluded that no recrystallization of the API took place during the processing of the lipid nanodispersions into ODFs.

### 3.3. Characterization of ODFs Containing API Particles

ODFs containing nano- or microparticles have already been discussed and characterized in literature. It was shown that nanoparticles are very well suitable for the embedding in ODFs. Due to their very small size, high API amounts could be realized with a good film quality [12,13]. To allow a good comparability of all films under investigation in this study, the particle-containing films were prepared with an API amount of 4 wt.%. Measurements of the API-content of the ODFs revealed loadings between 0.34 and 0.45 mg cm^−2^ for the micro and nanoparticle-loaded ODFs (Table 3) rather low FENO content of the nanoparticle-loaded ODFs could be explained by the deposition of unground particles in the stirred media mill, which slightly reduced the solids content of the nanosuspension. The high NAP content in the microparticle-loaded ODFs were assumed to be due to an irregular dispersion of the particles in the film matrix, which was also indicated by the high standard deviation.

**Figure 7:**
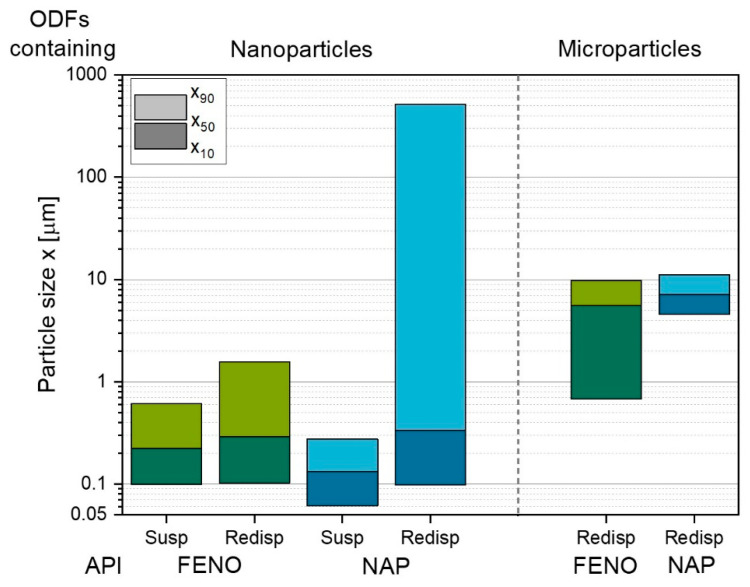
Particle sizes of nanosuspensions (susp) after milling of FENO and NAP and particle sizes after redispersion (redisp) of nano- and microparticle-containing ODFs.

After milling the poorly water-soluble APIs in a stirred media mill, nanosuspensions with mean values of 130 nm for NAP and 220 nm for FENO were obtained (Figure 7). These suspensions (susp) were used for the preparation of the nanoparticle-containing ODFs. To evaluate the effectiveness of the additives to prevent particle agglomeration in the formulation during milling, the ODFs were redispersed in a saturated FENO and NAP solution, respectively, and particle sizes were measured. Particle agglomeration was observed for both film formulations, as indicated by x_90_ values above 1 µm (redisp). Agglomeration was particularly evident among NAP-containing ODFs and was attributed to an insufficient homogenization of the particle in the HPMC solution. In previous studies with a similar particle formulation, agglomeration of the NAP particles did not occur when dispersed in the HPMC solution by using a stirred media mill or a dissolver [12].

The API microparticles embedded in ODFs were also redispersed in water and showed reasonable x_90_ values of approximately 10 µm for both film formulations, with broad particle size distribution for the FENO-containing ODFs.

### 3.4. Comparison of ODFs Manufactured by Different Formulation Strategies

To overcome the poor water-solubility of the model APIs used in this study, five different strategies were applied, and the films closer investigated in the sections above. To enable a comparability of the films formulated with different strategies, films with an API content of 4 wt.% were chosen for this comparison. For the lipid-containing ODFs, a total API-load of 4 wt.% could not be realized, so the maximum possible API content in the films was chosen for the comparison. Thus, the FENO-containing ODFs were loaded with 1.92 wt.% (MCT films, nanoemulsion) and 1.72 wt.% (tristearin films, nanosuspension), respectively. The NAP content in the lipid-containing films under investigation was 0.76 wt.% when MCT was used as lipid carrier and 1.33 wt.% when tristearin particles were the API carrier system. 

#### 3.4.1. Mechanical Film Properties

**Figure 8:**
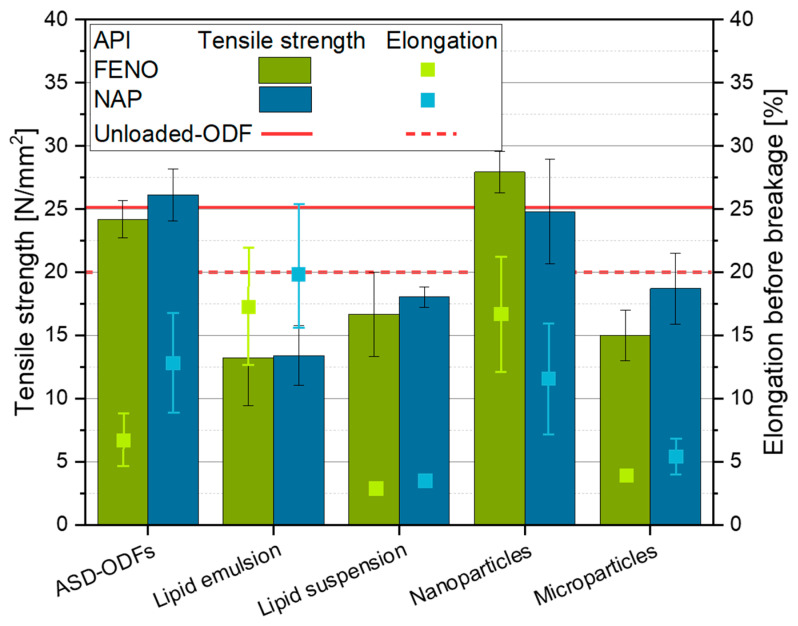
Tensile strength and elongation before breakage for API-loaded ODFs formulated with different strategies, n = 6.

When comparing the different formulation strategies, the mechanical film properties indicate the handleability of the films by a patient. Thus, the tensile strength was determined to describe the traction force that can be exerted before the films break, and the elongation before breakage to provide an estimate of the brittleness of the films compared with an unloaded-ODF as reference (Figure 8). 

Tensile strengths with a similar value as the unloaded-ODF were measured for ASD-ODFs. This indicates that the embedding of NAP and FENO into the film matrix as dispersed molecules did not significantly affect the tensile strengths of these films, although the FENO-loaded ASD-ODFs crystallized during the drying process. However, the elongation before breakage was lower than the unloaded-ODF for both API-loaded films. On the one hand, this could be attributed to the manufacturing process of the ASD-ODFs. HPMC was dissolved in a water/ethanol mixture before the API was added into the solution. Since HPMC is not soluble in pure ethanol, it can be assumed that the HPMC molecular chains are sterically differently arranged in these formulations based on their different interaction potential with a water/ethanol mixture. As a result, the elongation potential of the films decreased. In addition, differences in the elongation before breakage for the NAP- and FENO-loaded ODFs may be caused by the different states of the API molecules in the films. The studies presented above pointed out that NAP was molecularly dispersed in the film matrix, while FENO crystals were obtained for the film with 4 wt.%, resulting in more flaws in the contacts between HPMC molecules due to the predetermined breaking points at the contact between HPMC and API surfaces and thus a lower elongation of these films before breakage. 

For the films loaded with API nano- and microparticles, a dependency of the elongation before breakage on the particle sizes was found. It showed that the larger the particles embedded in the film matrix were, the lower was the film elongation before breakage. This confirmed that the size of flaws in the contact network of HPMC molecules increased and decreased both tensile strength and elongation, the larger the embedded particles were. While embedding nanoparticles in the film structure showed no significant influence on the tensile strengths compared to the unloaded-ODF (x_50_ = approximately 300 nm for both film formulations), the microparticles with a mean value of 5.5 µm for the FENO-containing ODFs and 7.1 µm for the NAP-containing films reduced the tensile strength by approximately 6 to 12 N mm^−2^, depending on the embedded API. 

Similar trends were observed when API-loaded tristearin particles were embedded in the film matrix. As shown above, the tristearin particles agglomerated during the film preparation (Figure 5), and particle sizes with a mean value of approximately 30 µm were measured. These films had a similar tensile strength as well as elongations before breakage as the films containing API microparticles. This showed that the influence of the size of the embedded particles was much stronger than the substance the particles were made of, if they were crystalline. 

Whenever API-loaded lipid emulsions where embedded in the film matrix, a different phenomenon was detected: the highest elongation before breakage, but the lowest tensile strengths compared to the other investigated films was found. The lipid droplets with a mean particle size of approximately 400 nm allowed a high flexibility of the films, which resulted in high elongations before breakage. However, these droplets cannot absorb the applied traction force, which weakened the films and resulted in the lowest tensile strengths.

#### 3.4.2. Specific Disintegration Time

**Figure 9:**
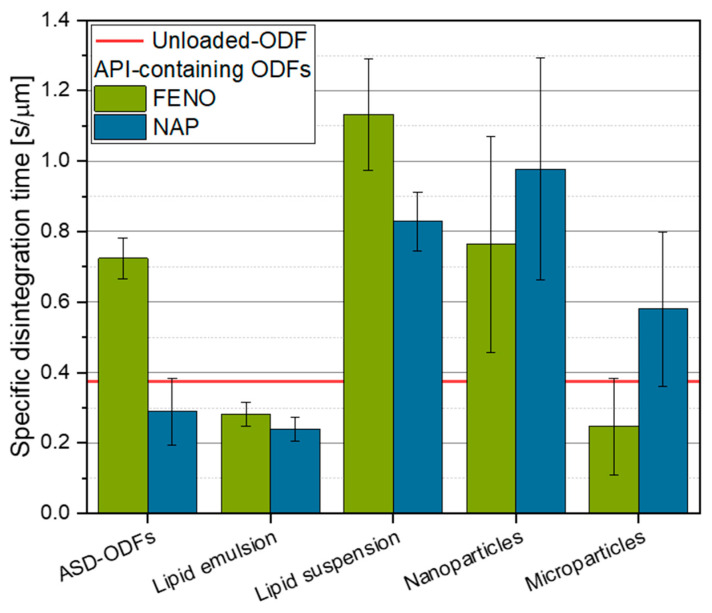
Influence of the film formulation strategy on the specific disintegration times of the FENO- and NAP-loaded ODFs, n = 4.

The disintegration time of ODFs in the mouth where only limited saliva is available is an important film property and could influence the patient compliance. Due to the slightly different film thicknesses, the measured disintegration times of the films referred to the film thickness and were given as specific disintegration times (Figure 9). The comparison of all films with an unloaded-ODF prove a strong dependency of the specific disintegration times on the film formulation strategy. The shortest disintegration times were measured for the films loaded with the API-containing lipid emulsions. These short specific disintegration times were assumed to be caused by the addition of the surfactant SDS to the formulation in order to stabilize the nanoemulsion during high-pressure homogenization. Previous studies already indicated that the addition of SDS in the ODF formulation reduced the film disintegration times by increasing the wettability of the film [35,36]. The results also indicated that embedding approximately 24 wt.% MCT droplets in the films did not negatively affect the disintegration time, as the specific disintegration time was shorter than that of the unloaded-ODF (Figure 9). The differences in the disintegration behavior seemed to be independent of the chemical properties of the loaded API. 

Significantly higher disintegration times were measured for the films containing the lipid nanosuspension. Although the particle formulation of the solid tristearin particles was similar to the MCT droplets (both were stabilized with SDS during high-pressure homogenization), the lipid content in these films was significantly higher at 49.6 wt.%, resulting in the longest disintegration times measured in this study. It should also be taken into account that tristearin forms platelet-like particles (in β-modification) during crystallization [32,37] which may further delay the disintegration of the films due to the particles’ large hydrophobic specific surface area. The longer disintegration times of the FENO-loaded ODFs is again in line with the higher lipophilicity of FENO (log P 5.2), which also causes higher loading capacity in lipid-based ODFs (Section 3.2) and lower maximum capacities in ASD-ODFs (Section 3.1) for FENO, respectively. Due to the crystalline structure of the lipid particles, the API molecules are assumed to be mainly located at the surface of the particles. Therefore, the hydrophobic properties could be an enhancing factor that further prolongs the wetting and, by that, the disintegration times of these films. Future in-depth studies can assist to confirm this theory.

A clear influence of the API on the specific disintegration times of the films was also shown for the ASD-ODFs. When 4 wt.% NAP was loaded in the HPMC matrix, the API was molecularly dispersed in the polymer (see Figure 2), resulting in short disintegration times. Embedding the same amount of FENO (4 wt.%) in the film-forming polymer resulted in the formation of API crystals. These led to a significant higher disintegration time of the FENO-containing ASD-ODFs. Again, the higher hydrophobicity of FENO contributes to this drastic difference in disintegration time of approximately 3:1 towards NAP. 

In general, studies indicate that the embedding nanoparticles into ODFs did not lead to higher disintegration times than for the unloaded-ODF, especially when the particle-containing films contained a surfactant and the unloaded-ODFs contained only the matrix polymer and a plasticizer [35]. However, the results obtained in this study show approximately twice the specific disintegration times for the nanoparticle-containing ODFs compared to the unloaded formulations and very high standard deviations. The long disintegration times are assumed to be caused by the agglomerates formed during film processing, as indicated by the elevated x_90_ values (Figure 7). In addition, the particle size measurements showed a wide particle size distribution in the films, which is assumed to cause the high standard deviations of these results, as these large hydrophobic particles may inhibit a uniform disintegration throughout the film. 

The embedding of API microparticles in the film matrix did not drastically alter the disintegration times compared with the unloaded-ODF but resulted in a macroscopically inhomogeneous surface of the ODFs, which was reflected in the high standard deviation of the results. Interestingly, the FENO-loaded films showed a clear trend to a quicker disintegration than the NAP-loaded films. Accordingly, the hydrophobicity of the API seems not to play an essential role in the wetting of films containing microparticles.

#### 3.4.3. Dissolution Behavior of API-Loaded ODFs

The aim of this study was to improve the bioavailability of the poorly water-soluble APIs NAP and FENO by using different formulation strategies and embedding these in ODFs. Whether this resulted in shorter dissolution times of the APIs compared to the ODF containing unformulated API micro particles was further investigated in this section. 

**Figure 10:**
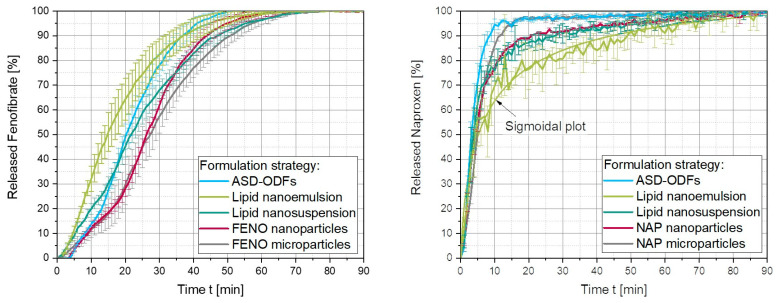
Dissolution behavior of FENO-loaded (**left**) and NAP-loaded (**right**) ODFs prepared by different formulation strategies, n = 6.

The dissolution behavior of the five FENO-loaded ODFs showed that the release of FENO could be accelerated when they were formulated in ASDs, embedded in a lipid matrix or ground into nanoparticles, compared to the released FENO from the microparticles-containing films (Figure 10, left). All films loaded with FENO showed a delay of approximately 2 to 4 min before the API was released. This delay was caused by the disintegration of the film before the API could be released in the medium. The fastest release was observed when API was formulated in a lipid nanoemulsion. Then, 80 % FENO was then released within 28 min. This correlated with the specific disintegration time, which was the shortest for the ODF containing the lipid emulsion (except for the microparticle-loaded ODF, Figure 9). Although the API molecules were assumed to be located, among others, inside the lipid droplets, the diffusion of the FENO molecules to the droplet surface seems to result in no significant delay during the dissolution process. Due to the large sizes of the MCT droplets, it could be excluded that lipids passed through the filter in the flow through cells during the experiment. 

The second fastest release of FENO (Q = 80 %) was from the ASD-ODFs within 31 min, which was faster than from the nanoparticle-loaded ODFs (37 min), with the same disintegration times. In this case, it is the hypothesis that although FENO recrystallized during the preparation of the ASD-ODFs, the overall specific API particle surface in the ASD-ODFs was higher, according to a huge aspect ratio of the FENO flakes discovered (e.g., Figure 2), than in the films containing FENO nanoparticles, resulting in shorter release times. As expected, the release times of the films loaded with FENO microparticles were the longest at 43 min, although the disintegration times of these films were the shortest (Figure 9). 

These measurements showed that the high lipid content of 50 wt.% in the ODFs formulated with FENO-loaded lipid nanosuspensions to increase the API contents in the films, had a negative impact on the disintegration time as well as the release of FENO from these ODFs. With 39 min (Q = 80 %), these films showed the longest release times among these different formulation strategies for poorly water-soluble APIs. Although the API molecules were assumed to be located at the particle surface, the wettability and the disintegration of the films are crucial for a good release of the API from the ODFs. 

In addition to the FENO-loaded ODFs, the dissolution behavior of the films loaded with NAP was also investigated (Figure 10, right). These films showed a completely different behavior than the FENO-loaded ODFs and the results must be considered critically as the low concentrations applied were closer to the limit of quantification. The manufacturer of the applied UV-Vis spectrophotometer states that the device measures the adsorption (A) with an accuracy of ±0.01 A. With an API load of 4 wt.%, the dissolution medium has a NAP concentration of approximately 4 mg L^−1^ after complete release, resulting in an adsorption of approximately 0.0284 A. Thus, the release data are within the range of the measurement accuracy. However, to account for the ODFs with a lower NAP loading, a control measurement was made with 1 mg L^−1^ NAP. This gave an adsorption of 0.0075 A, only deviating 5 % from the expected value when compared to a 10 mg L^−1^ NAP solution (0.071 A). Therefore, it is assumed that the tendency of the measured data is correct and can be used for comparison of the different formulation strategies. However, since the concentration in the lipid nanoemulsion films was even smaller (0.8 mg L^−1^ after complete dissolution), the measured concentration values fluctuate more pronounced. Therefore, for better comparability of the dissolution time at 80 % release, a sigmoidal plot was fitted to these data. 

Although the ASD and lipid emulsion films disintegrated significantly faster than the other formulations (e.g., Figure 9), only the dissolution time of the ASD film outperforms the dissolution of the microparticle film. This might be related to the higher hydrophilicity of NAP and thus lower log P value compared with FENO in combination with the low API concentrations in the dosage form, which needs to be further evaluated in future studies. The slower dissolution of the lipid suspension and nanoparticle film can be related to their slower disintegration times. Furthermore, the redispersed nanoparticles of NAP had a remarkable share of agglomerates, even much larger in x_90_ than for NAP microparticles (Figure 5). These results likewise highlight the importance of the disintegration time of ODFs for API dissolution behavior and give hints first towards the choice of the formulation strategy for ODFs loaded with poorly water-soluble APIs based on their log P value.

#### 3.4.4. Summarizing View on Formulation Strategies

**Figure 11:**
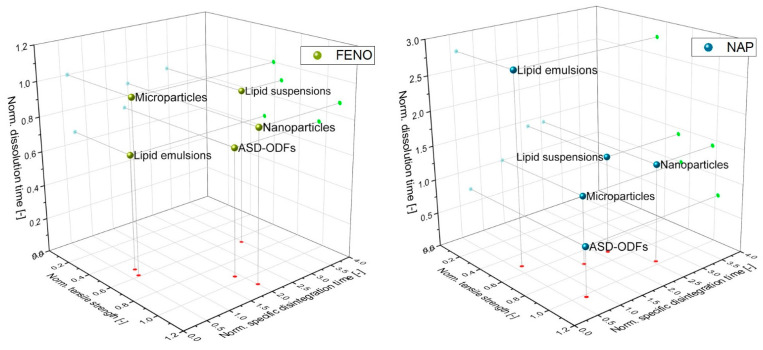
Comparison of the influence of film formulations with the normalized film properties tensile strength, specific disintegration time and dissolution time (Q = 80 %); FENO-containing ODFs (**left**), NAP-ODFs (**right**).

To finally allow a comprehensive evaluation of the different formulation strategies in regard to the film properties, the following three film properties were considered: tensile strength as representative of the mechanical film properties, specific disintegration time and dissolution time (Q = 80 %). In this consideration, all film properties were normalized (norm.) to the unloaded-ODF, except the dissolution times, which were normalized to the microparticle-loaded ODFs (Figure 11). 

Formulation of FENO in the ODFs show an improvement in dissolution time for all formulation strategies, as indicated by normalized specific dissolution times below one. The shortest FENO releases were observed when the API was embedded in the lipid emulsions as well as when ASDs were prepared. However, it was found that the film properties were affected by the different formulations. While the disintegration times of films containing lipid emulsions could be reduced (norm. specific disintegration time 0.62), their mechanical properties were deteriorated (norm. tensile strength 0.52). In this context, the ASD-formulations were clearly more advantageous when it came to the mechanical strength of the films (norm. specific disintegration time 0.75) but these films had significantly longer disintegration times (norm. disintegration time 1.90). Normalized tensile strengths above one could only be achieved for the nanoparticle-containing ODFs, but these films had disintegration times twice as high as the unloaded film and a normalized dissolution time of only approximately 0.88. In this context, the film in which the lipid suspensions were embedded performed worst. It had a high disintegration time, low mechanical strength and only a slight improvement in the release of FENO from the film. 

Comparing these results to the NAP-loaded ODFs, most films show similar behavior in terms of the tensile strength and the disintegration time. ASD-ODFs showed a significant improvement in film properties in terms of disintegration time, which was attributed to the molecular dispersion of the API in the film matrix. When the films were loaded with NAP, the ASD-ODFs clearly show the best results: the formulation strategy led to an improvement of the dissolution time, the normalized tensile strength of this film was higher than one and the disintegration time could be shortened (norm. specific disintegration time 0.75). 

In summary, the best results were obtained for the ODFs in which the lipid nanoemulsions were embedded and the ASD-ODFs, although sacrifices had to be made according to the film quality. Very good results were obtained for the ASD-ODFs in which NAP was embedded, while the more lipophilic FENO clearly prolonged the disintegration time of these films, and here the ODFs embedding lipid nanoemulsions proved to be an attractive alternative formulation strategy.

## 4. Conclusions

The two poorly water-soluble APIs FENO and NAP could be successfully formulated to orodispersible films by applying different enabling strategies for BCS II drugs. It was shown that the physicochemical properties of the API had an important role when the substances were formulated as ASDs or loaded in lipid nanocarriers. Thus, the formulation of NAP in the ASD-ODFs resulted in an API-load of up to 8 wt.% without any recrystallization, while the API amount was limited to 2 wt.% when FENO was formulated in the films, indicating different intermolecular bonds between the APIs and the film forming matrix. However, the higher lipophilicity of FENO, indicated by the log P value, had a positive effect when loaded to the lipid carriers: the loading capacity of FENO in the lipid nanocarriers was much higher for both types of lipids (nanosuspension and emulsion), than for NAP. 

The comparison of the film properties shows that each formulation has its strengths and weaknesses. It became clear, for example, that the shortest disintegration times could be realized with films formulated as ASDs or when lipid nanoemulsions were embedded in the film matrix. However, the ASD-ODFs show a lower brittleness than the films formulated with the lipid nanoemulsions and the embedded MCT nanoemulsion droplets significantly reduced the tensile strengths of the films. While the films containing nanoparticles had in general good mechanical properties, their disintegration times were much longer than for the other films. Finally, for FENO-containing ODFs, the disintegration times could be shortened for all formulations compared to the ODF with the micronized starting material. 

Due to the results of the presented systematic and comprehensive study, advantages and disadvantages for each solubility enhancing formulation strategy could be highlighted. By referring, e.g., to the lipophilicity of the APIs, general rational hints towards suitable enabling strategies for poorly-soluble APIs can be chosen. However, for the definition of an efficient and robust formulation guidance of ODFs with poorly soluble drugs, further elucidating studies are required to prove and quantify the correctness of the advice towards a formulation strategy for each drug/polymer combination.

## Figures and Tables

**Table 1:** General characteristics of fenofibrate and naproxen [23].

	Fenofibrate	Naproxen
Log P	5.2	3.2
Solubility in water (25 °C)	0.42 mg L^−1^	15.90 mg L^−1^
Molecular weight	360.8 g mol^−1^	230.3 g mol^−1^

**Table 2:** API content embedded in lipid dispersions and total API content in an ODF with a surface weight of 10 mg cm^−3^, n = 3.

	Nanoemulsion MCT	Nanosuspension Tristearin
API content embedded in lipid	API: FENO target: 4 wt.%/actual: 3.8 wt.% target: max./actual: 8.0 wt.%	API: FENO target: max./actual: 3.5 wt.%
	API: NAP target: max./actual: 3.2 wt.%	API: NAP target: max./actual: 2.7 wt.%
Content of lipid in ODF	24.1 wt.%	49.6 wt.%
API content in ODF	API: FENO 4 wt.% → 0.091 ± 0.002 mg cm^−2^ max. → 0.192 ± 0.001 mg cm^−2^	API: FENO max. → 0.172 ± 0.020 mg cm^−2^
	API: NAP max. → 0.076 ± 0.011 mg cm^−2^	API: NAP max. → 0.133 ± 0.002 mg cm^−2^

**Table 3:** Content of FENO and NAP in particle-containing ODFs, n = 3.

Particle Type	FENO Content in ODF [mg cm^−2^]	NAP Content in ODF [mg cm^−2^]
Nanoparticles	0.340 ± 0.056	0.425 ± 0.008
Microparticles	0.389 ± 0.004	0.451 ± 0.040

## Data Availability

The data presented in this study are available on request from the corresponding author.

## Supplementary Materials

The following supporting information can be downloaded at: https://www.mdpi.com/article/10.3390/pharmaceutics15010017/s1, Figure S1: Particle size after redispersion of ODFs with different contents of HCT nanoemulsions embedded in the film matrix.
