# Supplementary material for: Embedding of Poorly Water-Soluble Drugs in Orodispersible Films—Comparison of Five Formulation Strategies
Source: Pharmaceutics. 2022 Dec 21;15(1):17. doi: 10.3390/pharmaceutics15010017 (PMC9864024; doi:10.3390/pharmaceutics15010017)
Supplement: Supplementary file 1 [file pharmaceutics-15-00017-s001.zip › pharmaceutics-2084473-supplementary.pdf]

Supporting Information

for

Embedding of poorly water-soluble drugs in orodispersible films – Comparison of five formulation strategies

Denise Steiner [1,2,3], Marius Tidau [3,4] and Jan Henrik Finke [3,4]

1. Institut für Pharmazeutische Technologie und Biopharmazie, Technische Universität Braunschweig, Mendelssohnstraße 1, 38106 Braunschweig;
2. Current address: Universität Tübingen, Pharmazeutisches Institut, Pharmazeutische Technologie, Auf der Morgenstelle 8, 72076 Tübingen, Germany;
3. Technische Universität Braunschweig, Zentrum für Pharmaverfahrenstechnik (PVZ), Franz-Liszt-Straße 35a, 38106 Braunschweig;
4. Technische Universität Braunschweig, Institut für Partikeltechnik, Volkmaroder Straße 5, 38104 Braunschweig, Germany.

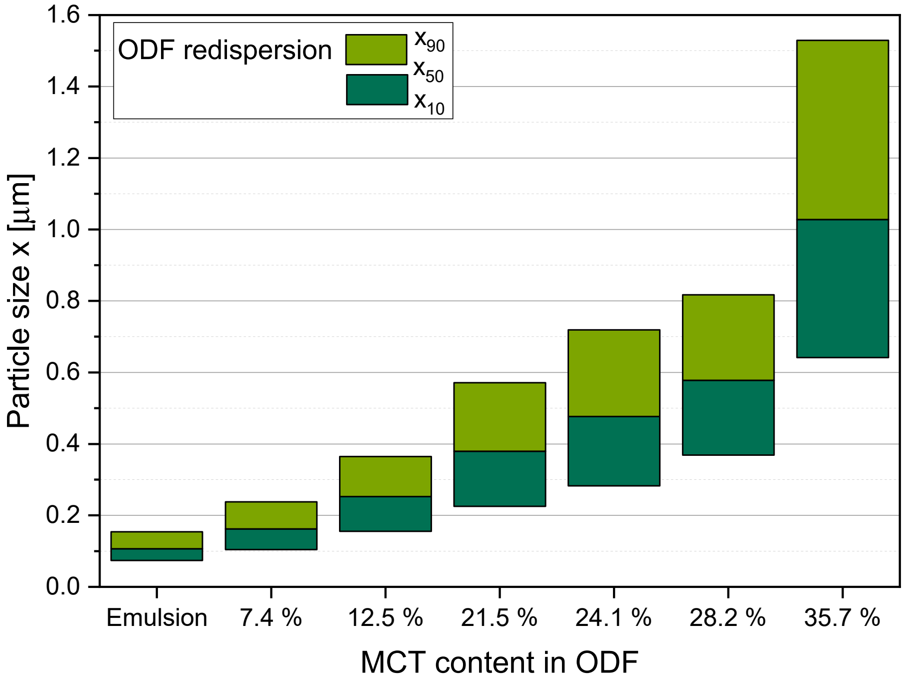

Figure S1. *Particle sizes after redispersion of ODFs with different contents of MCT nanoemulsions embedded in the film matrix.*

In order to enable a sufficient API load in the ODFs, a MCT content of 24.1 wt.% was chosen in this study.
